# Effect of Climatic Conditions and Water Bodies on Population Dynamics of the Dengue Vector, *Aedes aegypti* (Diptera: Culicidae)

**Published:** 2017-03-14

**Authors:** Shabab Nasir, Farhat Jabeen, Sadia Abbas, Iram Nasir, Mustapha Debboun

**Affiliations:** 1 Department of Zoology, Government College University, Faisalabad, Pakistan; 2 Department of Statistics, Government College University, Faisalabad, Pakistan; 3 Harris County Public Health and Environmental Services, Houston, TX, USA

**Keywords:** Population dynamics, Climatic conditions, Dengue vector, *Aedes aegypti*

## Abstract

**Background:**

The population of mosquitoes is mainly influenced by the biotic and abiotic factors. Although *Aedes aegypti* was reported until 1960’s in the Punjab, Pakistan, the population has increased dramatically since 2009 and caused severe epidemics in 2011 due to heavy floods and rains. Thus, this study was carried out to know the effect of biotic and abiotic factors on the population of *Aedes aegypti*.

**Methods:**

Mosquitoes were collected from fresh, sewage, and rain water ponds, fish ponds, rice fields, tyres, tree holes, and manmade storage containers present in and around residential homes twice during every winter (October–February), summer (March–June) and monsoon season (July–September) from marked rural areas.

**Results:**

More mosquitoes were collected in 2010 and 2011 due to floods than other years with heavy rains. High population (52.4%) was recorded during the rainy season due to high temperature (28–36 °C) and high relative humidity (up to 75%), while low population was recorded during the winter due to low temperature (< 5 °C) and low relative humidity (< 22%). Specimens were recorded indoors when outside temperature was below freezing point. *Ae. aegypti* was largely collected from tyres and urban areas mostly during the rainy season from small water containers. Years, months, seasons, temperature and relative humidity were statistically significant concerning the population dynamics of mosquitoes.

**Conclusion:**

Abiotic factors (temperature & relative humidity) along with habitat have significant impact on population dynamics of mosquitoes.

## Introduction

Mosquitoes act as vectors for many vector-borne diseases (Vaughn et al. 2009) such as malaria, dengue and yellow fever (Snow et al. 2005). They are found all over the world except in Antarctica (Gadahi et al. 2012). *Anopheles*, *Culex* and *Aedes* are three medically important genera of mosquitoes. *Aedes* mosquitoes are recognized as daytime biters, widely distributed in more than 100 countries of the world (Gratz 2004). *Aedes aegypti* and *Ae. albopictus* are two main mosquito species involved in dengue transmission to humans (Knudsen 1995). Dengue was a sporadic disease during the previous century. However, currently it has become a major health problem throughout the world due to the modern means of transportation, trade, population growth, mobility and environmental change. Twenty two thousand deaths are reported annually worldwide mainly in children and young adults (Haile-Mariam and Polis 2009, Vaughn et al. 2009).

Recent epidemic of dengue in Pakistan has been attributed to heavy rains and floods, deterioration of health, and sanitation principles at all stages of the community (Anonymous 2011). In 1994, dengue fever (DF) and dengue hemorrhagic fever (DHF) were first recorded in Karachi, Pakistan (Chan et al. 1994). In 2011, about two decades later from its first record in Pakistan, dengue appeared in its epidemic form in the Punjab province with 100 confirmed cases daily (Khawar 2011). The hotspot of the epidemic was the capital city Lahore where more than 131 people dead and over 12,000 people infected according to the Punjab Health Department (Anonymous 2011).

Mosquitoes have a well-adjusted life, particularly by inhabiting quiet pools, underground buildings, and see page areas near any bodies of water. Water bodies are different from each other in respect to chemical properties of water (acidity or alkalinity, freshness, salty or brackish) and the type or amount of vegetation present in them (Foster and Walker 2002). Thus, there is no doubt some mosquito species use more than one kind of habitat. However, generally many mosquitoes can be grouped according to their preference for water bodies such as permanent, flood, temporary or container water (Vanwambeke et al. 2007). Since dengue fever is a viral infection, and no vaccine or treatment is available for people, entomologists are key researchers who had better understand the vector and its life cycle to develop an integrated mosquito management program (Rathor 1996, Gratz 2004). The study of the biology of the vector along with the climatic conditions suitable for his life cycle is vital for entomologists who help in the timely application of mosquito management system against dengue vectors (Sharma et al. 2005). Container location and capacity, water source and temperature, all can vary seasonally. *Aedes aegypti* breeds in standing water generally in artificial containers. The amount of rainfall, temperature, and relative humidity are very important climatic factors that affect the population of *Aedes* mosquitoes especially *Ae*. *aegypti* (Scott et al. 2000).

Due to these reasons, we decided to explore the role of the climate, water bodies (size and location), physical and chemical properties of water, environmental abiotic factors and distance from human population on the population dynamics of *Ae. aegypti.*

## Materials and Methods

This study was conducted from 2009 to 2013 involving the collection of *Ae. aegypti* from rural and urban areas of the Punjab (31.0000° N, 72.0000° E and 139m above sea level) (Anonymous 2014) and identification of key ecological factors affecting the population dynamics and seasonal abundance of *Ae*. *aegypti*. Punjab is the most populous province with an area of 79,284 square miles. Almost 70% of its population consists of farmers who live in villages or around the big cities. Its boundaries meet with Jammu and Kashmir in the northeast, Indian Punjab and Rajasthan in the east, province Sindh in the south, Baluchistan and KPK (Khyber Pakhtunkhwa) in the west and Islamabad and Azad Kashmir in the north. It lies on the margin line of the monsoon climate. Its climate is mixed, some areas are too cold like Murree (up to −4 °C) and some are hot like Multan (up to 47 °C). The average annual precipitation is low, but sometimes-heavy rains occur due to monsoon airs during July–September that become the cause of heavy floods (Anonymous 2015).

Mosquitoes were collected randomly from forests, fields, parks, and residential areas (in and around the houses). Since *Ae*. *aegypti* prefers the small artificial containers, we collected from buckets, waste water near houses, seepage pools, dairy and poultry farms (Harrel et al. 2011), used tyres, broken vases, bottles, irrigation channels, cemented tanks, bamboo sticks (Bashar et al. 2005), tyre shops, and tree holes (Akram and Lee 2004). Adults were collected with aspirators and collection nets, while larvae and pupae with plastic dippers.

### Seasonal Abundance

**Fig. 1.**
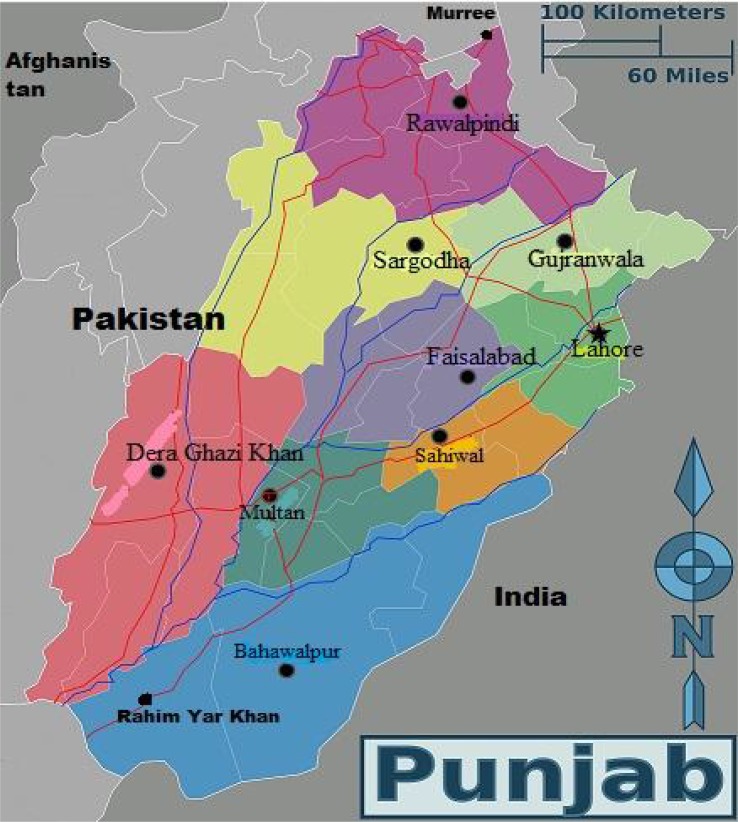
Location of selected districts of the Punjab for the collection of *Aedes* mosquitoes, 2009–2013

Collection (adults with aspirators and collection nets, while larvae and pupae with plastic dippers) was done seasonally to examine the population fluctuations in different areas of the Punjab, Pakistan (Fig. 1). Therefore, the year was divided into three quarters based on seasons, i.e. summer (March to June), rainy or monsoon (July to September) and winter (October to February) (Mohiuddin 2007).

Global positioning system (GPS) was used to record the height from sea level and coordinates (longitude and latitude) of marked sample sites during the survey. Temperature, relative humidity and pH were measured with a multi-meter (Elmetro Poland, Model # CPC 401) while the location of sites were recorded with Magellan GPS (Explorist, 660). After the adult mosquitoes were killed using the poison bottle, the genera were separated and counted at the site to identify the seasonal abundance (Reisen and Milby 1986). The larvae were placed in 500ml plastic jars filled with water from the source to keep them alive during transport to the Vector Biology Laboratory, Government College University and Faisalabad, Pakistan and reared in rearing cages. After the adults emerged they were killed, pinned, and identified to species level with the help of available taxonomic keys (Barraud 1934, Darsi and Pradhan 1990). Specimens were identified using the microscope (Nikon Binocular Microscope Eclipse, Model # E-100).

### Biotic and abiotic factors

Biotic (flora and fauna present in the water) and abiotic factors including the physical condition of water (clear, turbid, and foul), pH, temperature and relative humidity (RH) (Akram and Lee 2004, Harrel et al. 2011) have been sampled from each site. We also recorded the location of each site as urban or rural and the distance of each site from the residential areas. After ANOVA, chi-square test was applied on significant factors and then logistic regression was applied on highly significant factors effecting the population.

### Statistical analysis

We used Mathematica 7 to analyze the data for chi-square value to determine the relationship between different variables affecting the population of *Ae. aegypti.* Logistic regression model was also used for significant factors from chi-square test to identify the key factors affecting the population of *Ae. aegypti.* A stepwise forward approach was applied, for this model (Vanwambeke et al. 2007).

## Results

*Aedes aegypti* mosquitoes were collected from different rural and urban areas of the Central Punjab (Lahore, Faisalabad, Sargodha, Sheikhupura and Gujranwala) and Upper Punjab (Rawalpindi, Islamabad and Murree) but not from Northern Punjab (Multan, Dera Ghazi Khan, Bahawalpore and Rahim Yar Khan). They were mostly sampled from latitude (max 033° 45.403, min 29° 57.859) and longitude (max 074° 45.403, min 072° 57.859) with elevation (max 1218m, min. 144m). Maximum population was collected from Lahore during the rainy season and minimum from Faisalabad during the winter season.

### Populations of *Ae. aegypti*

From 810 water container sites, 83 sites (10.25%) were positive for *Aedes* mosquitoes. Populations of *Ae. aegypti* showed a great variation in their density during all seasons (P-value= < 0.001). Maximum population (52.4% of the total collected population) was collected during the rainy season followed by the summer season (41.7%) while the lowest percentage (5.9%) was found during the winter. Similarly, maximum population (42.7%) was recorded during 2011 due to heavy rains followed by 35% in 2010, likely due to heavy floods in the province. Water quality had significant influence on population density (P-value< 0.001). Most populations were collected from turbid water (46.1%) followed by turbid foul (33.8%), clear (17.4%) and clear foul (2.7%). In addition, most of the population (97.8%) was found in standing water than in flowing water (2.2%). Color of the water was also significant (P= 0.04) in relation with population density since most of the population of *Ae. aegypti* was found in colorless medium (29.6%). In addition, light had a significant (P< 0.002) impact on population density with 60.0% population exposed in shady medium (60.0%) (Table 1).

**Table 1.** Key parameters for the presence of *Aedes aegypti* in Punjab area, Pakistan, 2009–2013

**Characteristics**	**Population of *Aedes aegypti***						**P-value**
	**2009**	**2010**	**2011**	**2012**	**2013**	**% age**	
**Seasons**							**˂0.001**
Winter	5	7	12	7	6	5.9	
Summer	37	61	72	46	41	41.7	
Rainy	49	75	92	57	52	52.4	
**Habitat**							**˂0.001**
Tyres	38	66	79	49	44	44.8	
Water bodies	18	24	29	19	17	17.4	
Others (Tree holes, cans, tanks and any other)	35	53	66	42	38	37.8	
**Water quality**							**˂0.001**
Clear	18	23	30	19	17	17.4	
Turbid	41	67	81	51	46	46.1	
Turbid foul	30	49	59	37	33	33.8	
Clear foul	2	4	5	3	3	2.7	
**Water condition**							**0.100**
Standing	89	141	169	108	97	97.8	
Flowing	2	2	5	2	2	2.2	
**Light**							**˂0.002**
Exposed	27	43	59	35	31	31.6	
Exposed shady	57	87	101	66	60	60.0	
Shady	7	13	14	9	8	8.4	
**pH**							**0.370**
≤ 7.00	0	0	0	0	0	0	
7.01–8.00	15	34	43	25	22	22.5	
8.01–9.00	57	79	93	62	56	56.2	
˃9.01	19	30	38	23	21	21.3	
**Area size (m)**							**˂0.001**
≤ 1.00	55	78	89	60	54	54.4	
1.01–10.00	3	6	8	5	4	4.1	
10.01–100.00	2	3	4	2	2	2.2	
˃ 100	31	56	73	43	39	39.3	
**Location**							**0.019**
Urban	67	106	146	86	78	78.1	
Rural	24	37	28	24	21	21.9	
**Temperature (°C)**							**˂0.001**
≤ 10	3	8	12	6	6	5.6	
11–20	3	10	29	11	10	10.3	
21–30	20	35	60	31	28	28.2	
31–40	63	87	70	59	53	53.9	
˃41	2	3	3	3	2	2.0	
**Relative humidity (%)**							**˂0.001**
≤ 30	4	4	5	4	3	3.2	
31–40	20	33	40	25	22	22.8	
41–50	29	47	67	39	35	35.0	
˃51	38	59	62	42	39	39.0	
**Distance from houses (m)**							**0.160**
≤ 25	72	109	135	85	77	77.5	
26–100	18	31	34	22	20	20.3	
˃100	1	3	5	3	2	2.2	

**Fig. 2.**
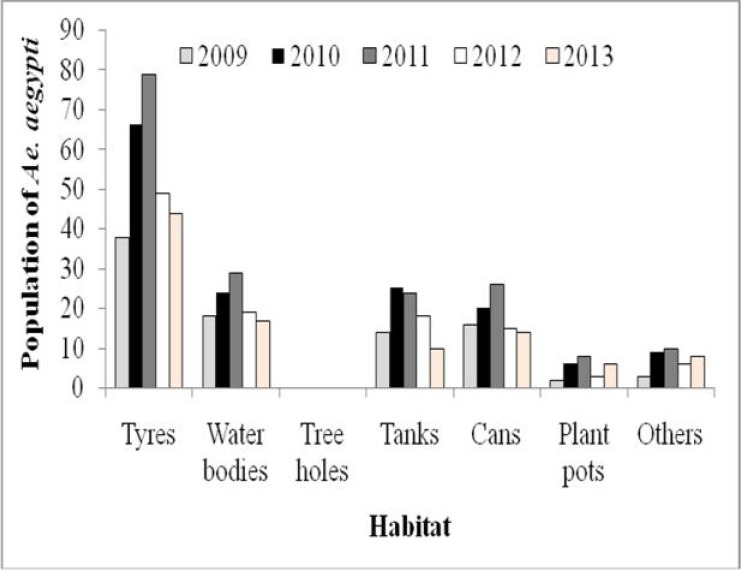
Number of *Aedes aegypti* collected from different visited habitats in Punjab, 2009–2013

There were more breeding sites during 2010 and 2011 than other years due to more rainfall and floods. *Aedes aegypti* was mostly collected from small containers such as buckets or cans and used tyres near or around residential areas (Fig. 2).

**Fig. 3.**
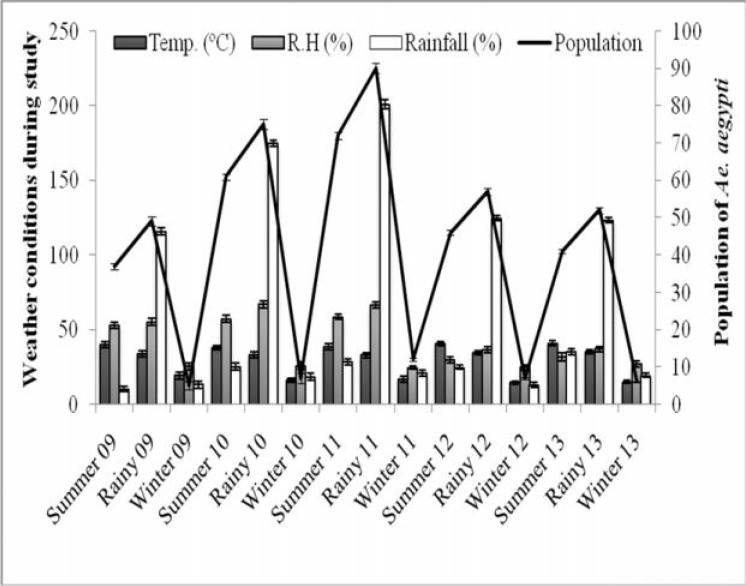
The relationship between abiotic factors during different seasons from 2009–2013 and the population of *Aedes aegypti*

Abiotic factors had a significant impact on the population of *Ae*. *aegypti.* More rain fell in 2011 resulting in an increase of its population. Thus, more rainfall in 2011, heavy floods in 2010, and the increase of the relative humidity in these years resulted in an increase of the population of *Ae. aegypti* and more numbers were collected (Fig. 3). In addition, more breeding sites were observed during the rainy seasons, particularly in 2010 and 2011. A little effect was observed on the population dynamics of *Ae*. *aegypti* because it was mostly collected from artificial water containers that were mostly found in the residential areas (Fig. 2).

### Habitat of *Ae. aegypti*

The majority of *Ae*. *aegypti* population was sampled in artificial containers (44.8% in tyres, 37.8% in tanks and cans). The remaining 17.4% has been collected in natural containers such as water bodies and none from natural tree holes (Fig. 2). Locality also showed a significant effect on the population level (P= 0.019). The population was recorded from urban areas (78.1%) than rural (21.9%). Among the flora, algae, grass, spirogyra, dragonflies, damselflies, frog tadpoles, and water bugs were recorded but we did not assess their role.

### Logistic regression analysis

The seasons had a significant role in *Ae. aegypti* population dynamics as seen in the rainy season (OR: 0.287, P= 0.003) than the summer (OR: 0.052, P= 0.005). Location also had a significant effect on the future population dynamics of this mosquito as found in the rural environment (OR: 0.496, P= 0.048) (Table 2).

**Table 2.** Logistic regression as predictor for the presence of *Aedes aegypti*, Punjab, 2009–2013

**Variables**	**Odds Ratio (OR)**	**95% confidence interval of OR**		**P-value**
		**Lower**	**Upper**	
**Seasons**				
Summer	0.052	0.069	0.437	0.005
Rainy	0.287	0.195	0.798	0.003
**Light**				
Exposed	0.593	0.197	1.899	0.395
Exposed Shady	0.292	0.101	0.865	0.33
Turbid Water	0.998	0.998	1.454	0.067
**Location**				
Rural	0.496	0.259	1.106	0.048
**Distance from houses**				
≤ 25	8.097	2.098	15.90	0.001
26–100	16.097	3.978	78.09	0.012

## Discussion

During this study, the population of *Ae*. *aegypti* was collected from 83 sites (out of 810) and showed a great variation during the entire year with the majority collected during the rainy season rather than the summer one. Small breeding habitats around or inside the residential areas, old tyres and exposed shady water pools were the sites chosen and preferred by *Ae. aegypti*. Water temperature ranging from 21 to 38 °C and a relative humidity higher than 35% supported the population density. However, the maximum population density was observed at 32–38 °C with relative humidity of about 50%. *Aedes aegypti* population was generally more supported in the urban environment than the rural one. Other scientists who also studied the distribution patterns according to seasons had similar results as ours showing that the cool season put a greater stress on the survival of adults and larvae than on the eggs (Preechaporn et al. 2007). Population density was flexible throughout the year depending on the larval breeding habitats.

Rainfall plays an important role in the population density of *Aedes* (Ndiaye et al. 2006). Our results showed that *Aedes* mosquitoes were collected during the rainy as well as summer season and these circumstances were mostly found in areas where the elevation was higher and ecological conditions were mild (Alto and Juliano 2001, Preechaporn et al. 2007). In Bangladesh, mosquitoes were collected from all sampling sites in the rural areas (Bashar et al. 2005). The domestic water storage containers (drinking water jars, buckets, and tanks) followed by tyres were the regular productive containers in our study area. The number of containers primarily filled with rainwater increased during the rainy season, generally in the used tyres. Therefore, this is a possible reason why *Ae. aegypti* population did not reach low levels at a time when rainfall was limited. As the people manage these containers, they also manage the production of mosquitoes in these containers unintentionally. These results are in agreement with the results of previous studies (Koenraadt et al. 2004, Barbazan et al. 2008). Mainly, a few key types of containers play important role for a large proportion of the pupal and adult production (Koenraadt et al. 2004, Barbazan et al. 2008).

However, the control measures such as placement of lids on water containers, use of larvicides or biological control agents, removal of discarded and unused containers have decreased the mosquito population density. Container capacity, water temperature, source of water, and container location, all of which could vary seasonally (Koenraadt et al. 2004, Kay and Nam 2005) have been reported as key ecological factors affecting population of *Ae. aegypti*. A study in Samoa Island revealed that the most productive containers during the dry and wet seasons were the buckets and used tyres (Lambdin et al. 2009). During this study, they also noted that the knowledge of environmental variables such as habitat density, water source, seasonal variations and neighborhood conditions played a significant role in container productivity. The typical rainfall system in the Punjab, normally during July–August was associated with a peak of *Aedes* population.

A better understanding of the environmental factors affecting the water container productivity is a key part of adaptive vector control efforts (Scott and Morrison 2008). Work is being carried out in tropical regions to investigate the effect of source reduction on the population of *Ae. aegypti* (Arunchalam et al. 2010). We can understand the population dynamics of *Ae*. *aegypti* more consistently and design a better effective vector control program by integrating the knowledge of breeding site density and the ecology of larval breeding sites.

## Conclusion

Environmental factors and breeding sites had a strong influence on the population dynamics of *Ae. aegypti* mosquito. Although the population was recorded during the entire year but most of the population was recorded during the rainy season. In addition, man’s activities had a strong relationship with the mosquito population.
